# Enzymatic basis of the Fc-selective intra-chain disulfide reduction and free thiol content variability in an antibody produced in *Escherichia coli*

**DOI:** 10.1186/s12934-022-01892-4

**Published:** 2022-08-19

**Authors:** Tomasz K. Baginski, Karthik Veeravalli, Rebekah McKenna, Christopher Williams, Katherine Wong, Christina Tsai, Daniel Hewitt, Karthik Mani, Michael W. Laird

**Affiliations:** 1 Protein Analytical Chemistry, Genentech, Inc., 1 DNA Way, South San Francisco, CA 94080 USA; 2 Cell Culture and Bioprocess Operations, Genentech, Inc., 1 DNA Way, South San Francisco, CA 94080 USA; 3 Purification Development, Genentech, Inc., 1 DNA Way, South San Francisco, CA 94080 USA; 4 Present Address: Adverum Biotechnologies Inc., 100 Cardinal Way, Redwood City, CA 94063 USA; 5 Present Address: ADC Therapeutics, 1510 Fashion Island Blvd, San Mateo, CA 94404 USA

**Keywords:** Antibody, Disulfide bond reduction, Free thiols, Fc glycosylation, Disulfide bond isomerase C (DsbC), Homogenate hold

## Abstract

**Background:**

*Escherichia coli* (*E. coli*) is a promising host for production of recombinant proteins (including antibodies and antibody fragments) that don’t require complex post-translational modifications such as glycosylation. During manufacturing-scale production of a one-armed antibody in *E. coli* (periplasmic production), variability in the degree of reduction of the antibody’s disulfide bonds was observed. This resulted in variability in the free thiol content, a potential critical product quality attribute. This work was initiated to understand and prevent the variability in the total free thiol content during manufacturing.

**Results:**

In this study, we found that the reduction in antibody’s disulfide bonds was observed to occur during homogenization and the ensuing homogenate hold step where in the antibody is exposed to redox enzymes and small molecule reductants present in homogenate. Variability in the downstream processing time between the start of homogenization and end of the homogenate hold step resulted in variability in the degree of antibody disulfide bond reduction and free thiol content. The disulfide bond reduction in the homogenate is catalyzed by the enzyme disulfide bond isomerase C (DsbC) and is highly site-specific and occurred predominantly in the intra-chain disulfide bonds present in the Fc C_H_2 region. Our results also imply that lack of glycans in *E. coli* produced antibodies may facilitate DsbC accessibility to the disulfide bond in the Fc C_H_2 region, resulting in its reduction.

**Conclusions:**

During *E. coli* antibody manufacturing processes, downstream processing steps such as homogenization and subsequent processing of the homogenate can impact degree of disulfide bond reduction in the antibody and consequently product quality attributes such as total free thiol content. Duration of the homogenate hold step should be minimized as much as possible to prevent disulfide bond reduction and free thiol formation. Other approaches such as reducing homogenate temperature, adding flocculants prior to homogenization, using enzyme inhibitors, or modulating redox environments in the homogenate should be considered to prevent antibody disulfide bond reduction during homogenization and homogenate processing steps in *E. coli* antibody manufacturing processes.

**Supplementary Information:**

The online version contains supplementary material available at 10.1186/s12934-022-01892-4.

## Background

Therapeutic antibodies are predominantly produced in mammalian cells, most commonly in Chinese Hamster Ovary (CHO) cells. Another production host, albeit not as common as CHO, is *Escherichia coli* (*E. coli*), which is capable of high-level expression and assembly of full-length IgG antibodies in the periplasm [1], however, *E. coli* lacks the enzymatic machinery to support post-translational modifications such as glycosylation. Therefore, antibodies produced in *E. coli* lack Fc effector functions, which could be advantageous when the intended therapeutic indication requires only an antibody’s blocking function. Depending on the intended molecule’s design (e.g., antibody fragments), use of *E. coli* as an expression system may offer some advantages over CHO cells. *E. coli* processes also have faster development timelines and lower production costs.

**Fig. 1:**
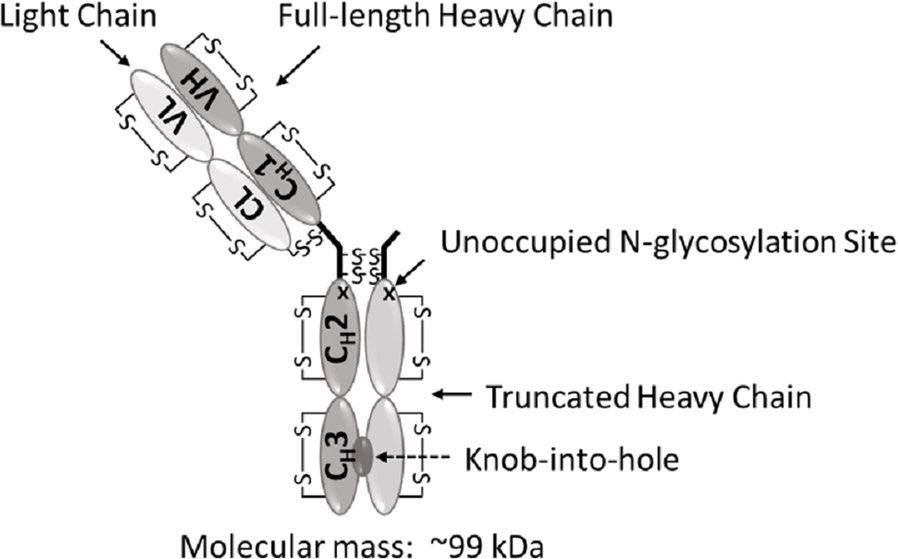
Schematic structure of the monovalent mAb1

In this work, an *E. coli* host with periplasmic expression was used for manufacturing-scale production of the unique recombinant “one-armed” monovalent mAb1, which has an ability to block the target receptor without inducing its dimerization and activation. In comparison, the standard format, i.e., a bivalent antibody with the same specificity activates the target receptor leading to the opposite effect than intended. mAb1 is a humanized, monovalent, monoclonal antibody based on the IgG1 framework. It is composed of a full-length heavy chain, a light chain, and a truncated heavy chain that consists of only the hinge region and the C_H_2 and C_H_3 domains (Fig. 1). mAb1 is aglycosylated (since it’s produced in *E. coli*) and has an approximate molecular mass of 99 kDa. To ensure proper heterodimerization of the Fc, where a full-length heavy chain is disulfide-linked to a truncated heavy chain, two heavy chains have been engineered utilizing the “knobs-into-holes” approach [2].

Proper assembly of mAb1 relies on the correct and efficient formation of multiple disulfide bonds. In mAb1, there are 22 cysteine residues, and all these cysteine residues are involved in disulfide bond formation resulting in 11 disulfide bonds, of which 3 are inter-chain and 8 are intra-chain linkages (Fig. 1). To promote disulfide bond formation, mAb1 was co-expressed with *E. coli* thiol-disulfide oxidoreductases DsbA [3] and DsbC [4], which are periplasmic enzymes responsible for disulfide bond formation and isomerization, respectively. Low levels of free thiols (unpaired cysteine residues) have been observed in naturally occurring IgG antibodies [5, 6] and free thiols are typically present in monoclonal antibodies produced in CHO cells [7, 8]. In antibodies produced in *E. coli*, little is known about the consistency of disulfide bond formation during the manufacturing-scale production and levels of free thiols in the resulting drug substance (DS) materials. As a result, the total free thiol content, a potential critical product quality attribute, was one of the attributes of particular interest during the development of mAb1 production process.

Here, we describe the selective intra-chain disulfide bond reduction in the Fc C_H_2 region and variability of total free thiol content observed during production of mAb1 in *E. coli*. The enzyme DsbC, which is over-expressed during the production of mAb1 for promoting disulfide bond isomerization, selectively reduced the Fc C_H_2 disulfide bond during homogenate hold step in downstream purification. Variability in free thiol content occurred due to differences in hold time of the homogenate derived from *E. coli* cells producing mAb1. Lack of the Fc glycosylation of mAb1 is an apparent cause of its susceptibility to free thiol formation in the Fc region. Lastly, we suggest a potential mechanistic explanation for the reduction of disulfide bonds in the Fc C_H_2 domain by DsbC.

## Results

### Variability of the total free thiol content during the development and manufacturing-scale production of mAb1

**Fig. 2:**
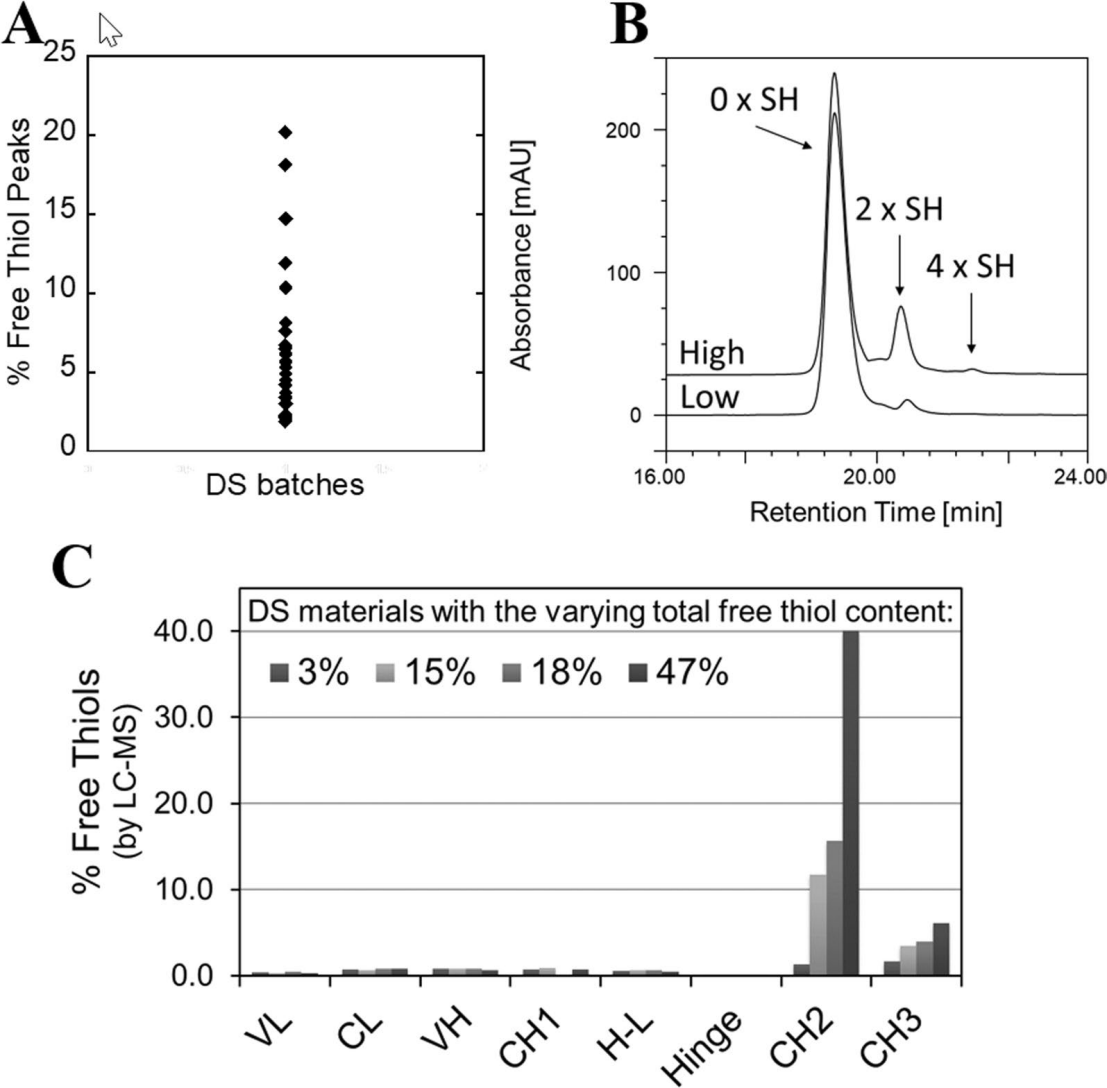
Free thiol content in mAb1. **a** Variability of the total free thiol content of the mAb1 DS batches produced at manufacturing-scale (MSS, MabSelectSure; AEX, Anion exchange, CEX, cation exchange). **b** Example chromatograms of mAb1 with the “Low” and “High” total free thiol content analyzed by RP-HPLC with the free thiol derivatization and UV detection (CUFDF, conditioned UFDF). **c** Site-specific levels of free thiols in mAb1 DS materials during development with the varying total free thiol content. Results shown are for n = 1 sample

During technical development of mAb1 production process, the total free thiol content (exposed and buried free thiols) was routinely determined using an in-house developed reversed-phase high performance liquid chromatography (RP-HPLC) assay, with the free thiol derivatization under denaturing conditions and UV detection, capable of separating and quantitating free thiol-containing mAb1 variants [9]. Using this assay, a high variability of the total free thiol content (up to ~ tenfold difference in % Free Thiol Peaks) was observed among various mAb1 drug substance (DS) batches produced at manufacturing-scale (Fig. 2a). This variability in total free thiol content, a potential critical product quality attribute, was highly undesirable for mAb1 production process. Thus, it prompted the detailed analytical characterization of mAb1 free thiols to identify process step(s) responsible for the free thiol content variability, and mechanism(s) involved in free thiol formation, with the overall goal of improving process control over the total free thiol level content.

### Analytical characterization of mAb1 free thiols

Free thiol analysis by RP-HPLC of mAb1 DS materials with varying total free thiol content indicated that the increased total free thiol content was predominantly due to the increased presence of mAb1 species containing 2 unpaired cysteine residues i.e., an equivalent of one disulfide bond, and to lesser extent species containing 4 unpaired cysteine residues (Fig. 2b). These results demonstrate that incomplete formation, reduction, or degradation of predominantly a single disulfide bond may have resulted in the increased total free thiol content of mAb1 materials.

To determine the main sites of free thiol formation, site-specific quantitation by LC–MS peptide mapping with differential isotope tagging of free thiols was performed. Analysis of developmental DS materials with the varying total free thiol content (3–47% Free Thiol Peaks) demonstrated that predominantly the intra-chain cysteine residues in the Fc C_H_2 domain corresponded to the free thiol sites (Fig. 2c). Intra-chain cysteine residues in the C_H_3 domain were the second location where the increase of free thiols was observed, but overall levels were significantly lower compared to the highest levels observed in the C_H_2 domain. Low levels of free thiols were also found for all other intra-chain disulfide bond locations and no apparent free thiols were detected at the inter-heavy-chain disulfide bonds.

### Identification of the process step responsible for mAb1 free thiol formation

**Fig. 3:**
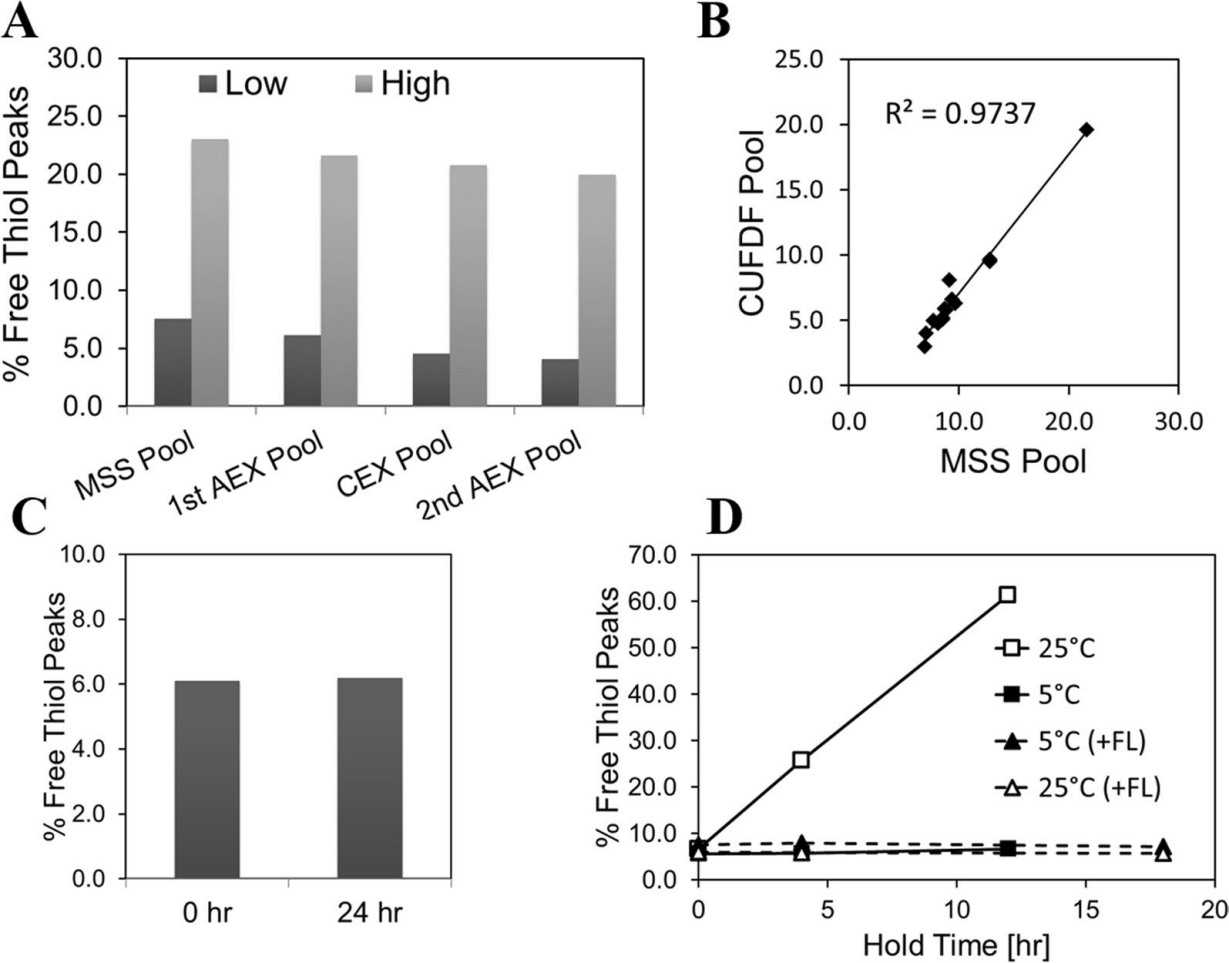
Effect of process parameters on total free thiol content in mAb1. **a** Clearance of mAb1 free thiols across 4 chromatography steps. Example of materials with low (Low) and high (High) total free thiol content are shown. **b** Correlation of the total free thiol content of MSS pools and corresponding CUFDF pools. **c** Effect of whole cell broth hold at the end of production culture step on total free thiol content. **d** Effect of temperature and presence of the flocculant (FL) on total free thiol content during the homogenate hold step. Results shown are for n = 1 sample

Having determined the location of mAb1 free thiols, we then set out to identify the process step(s) responsible for their formation. The following major steps constituted the mAb1 production process: production culture, harvest, and downstream purification. Production culture process parameters were controlled at target for the manufacturing-scale runs with significant variability in free thiol content as shown in Fig. 2a. No correlations were observed between upstream process performance and free thiol content. Downstream removal of free thiols across the four chromatography steps was low (Fig. 3a). Supporting this observation, the free thiol content in MabSelect SuRe (MSS) affinity chromatography pools (first step of the purification process) correlated well with conditioned ultrafiltration and diafiltration (CUFDF) pools (last step of the purification process) (Fig. 3b). These results indicate that the free thiol content variability observed in the DS materials was already present in MSS pool and that the downstream purification is not responsible for the variability of total free thiol content in mAb1.

During the harvest step of mAb1, the *E. coli* whole cell broth at the end of production culture is cooled down (approximately to 10 °C) and mAb1 is released from cells by homogenization. The resulting homogenate is clarified from the cellular contaminants using heat-assisted flocculation with a cationic flocculant, polyethyleneimine (PEI), followed by centrifugation prior to downstream purification. At the end of fermentation but prior to the cool-down, holding the whole cell broth for 24 h at 25 °C did not impact free thiol content (Fig. 3c). However, when the homogenate was held at 25 °C, the total free thiol content of mAb1 readily increased in a time-dependent manner (Fig. 3d). Addition of a flocculant to homogenate and/or decreasing the temperature to 5 °C during the hold step prevented free thiol formation during the homogenate hold. While whole cell broth hold or the homogenate hold steps are not part of routine manufacturing operations, these hold steps are sometimes necessary to accommodate operational flexibility.

**Fig. 4:**
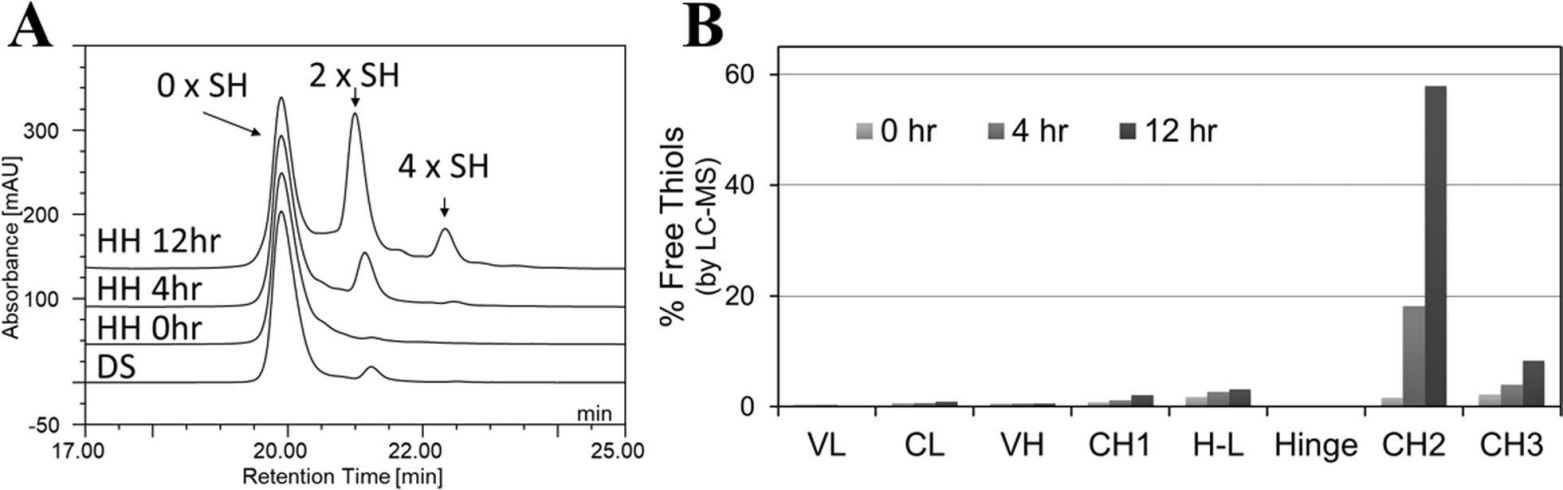
Effect of homogenate hold (0, 4, and 12 h hold) on **a** Total free thiol content in mAb1 MSS pools, **b** Site specific free thiol content of mAb1 MSS pools. HH (homogenate hold), DS (manufacturing-scale produced drug substance with minimal homogenate hold step (< 1 h)). Results shown are for n = 1 sample

Next, we investigated whether free thiols induced by the homogenate hold were similar in nature to the free thiols in mAb1 DS materials. Free thiol analysis by RP-HPLC of mAb1 after homogenate hold (0, 4, and 12 h at 25 °C), demonstrated that the homogenate hold produced the same pattern of free thiols species that was present in DS, namely the single open disulfide and to a lesser extent, the double open disulfide forms (Fig. 4a). Site-specific free thiol analysis showed that major free thiol sites matched those in the DS materials and the increased total free thiol content corresponded to the increase of free thiols predominantly in the Fc C_H_2 domain, and to a much lesser extent in the Fc C_H_3 domain (Fig. 4b). Altogether, these data demonstrated that disulfide bond reduction of mAb1 can occur during the harvest operations, specifically during the extended homogenate hold, resulting in free thiol formation at the same sites as in the DS materials.

### Determination of the enzymatic basis of free thiol formation in *E. coli* homogenate

**Fig. 5:**
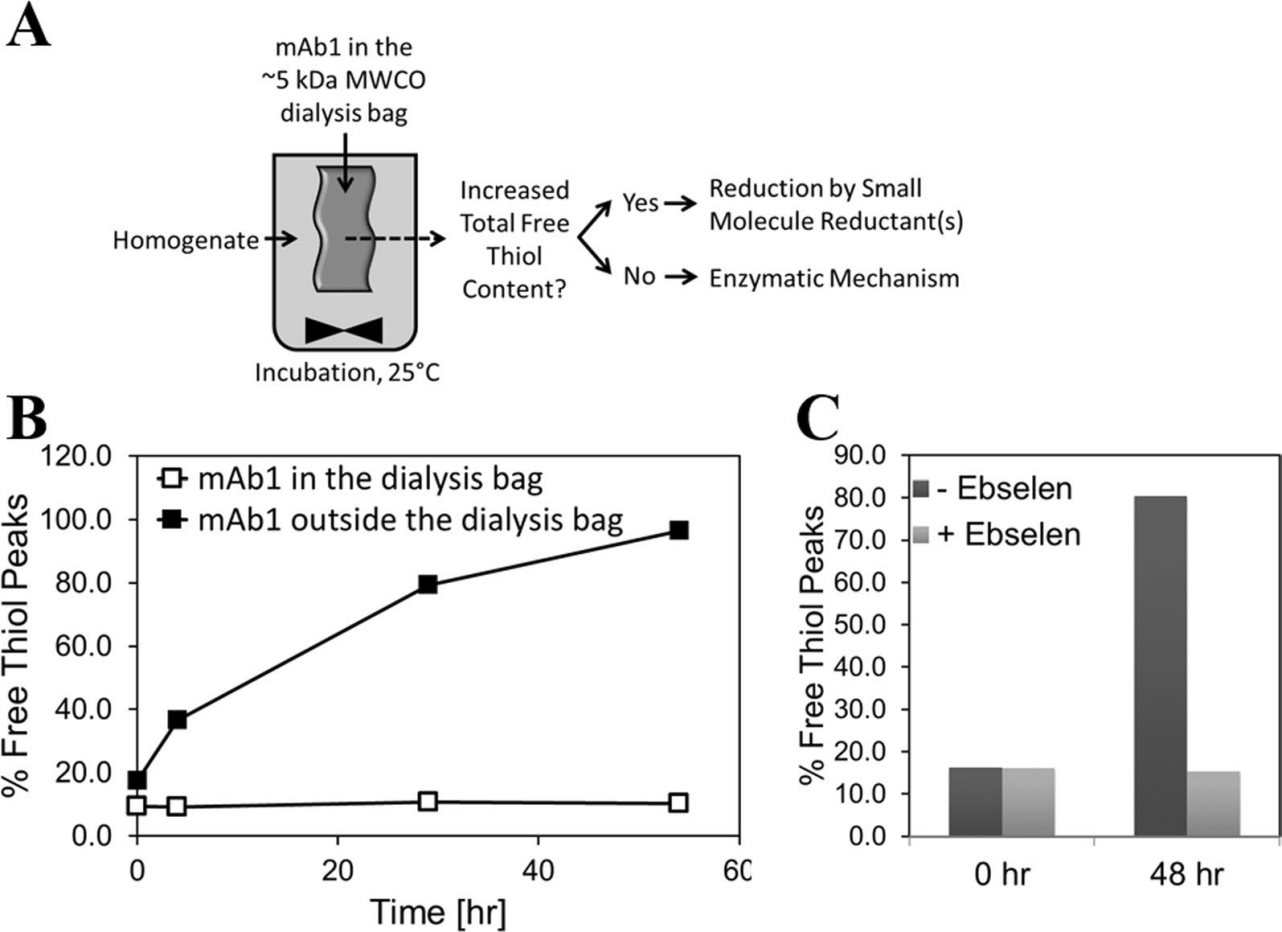
Identification of enzymatic basis for free thiol formation. **a** Dialysis experimental set up, **b** Free thiol formation with mAb1 inside or outside the dialysis bag (positive control), **c** Free thiol formation in the presence or absence of ebselen, a known inhibitor of *E. coli* thioredoxin reductase. Results shown are for n = 1 sample

In *E. coli* homogenate, mAb1 disulfide bonds can be reduced by small molecule (e.g., glutathione) and/or enzyme reductants. A dialysis experiment was performed to determine whether disulfide bond reduction in mAb1 was catalyzed by an enzyme or a small molecule. mAb1 MSS pool was placed inside the dialysis bag with the molecular weight cut-off (MWCO) of ~ 5 kDa and the dialysis bag was incubated in the *E. coli* homogenate derived from a fermentation that is not producing mAb1, for up to 54 h at 25 °C (Fig. 5a). Selected MWCO of the dialysis membrane was expected to allow for a free access of small molecule reductants from the homogenate to mAb1 MSS pool in the dialysis bag, while restricting access of any of the native *E. coli* enzymes with molecular weights greater than 5 kDa present in the homogenate. mAb1 MSS pool material spiked directly into the homogenate (outside the dialysis bag) served as a positive control for the free thiol formation.

Free thiols did not increase in mAb1 incubated inside the dialysis bag (Fig. 5b). As expected, free thiols increased in time-dependent manner in mAb1 spiked directly into the homogenate. These results indicate that the disulfide bond reduction in mAb1 during the homogenate hold step is likely catalyzed by an enzyme.

**Fig. 6:**
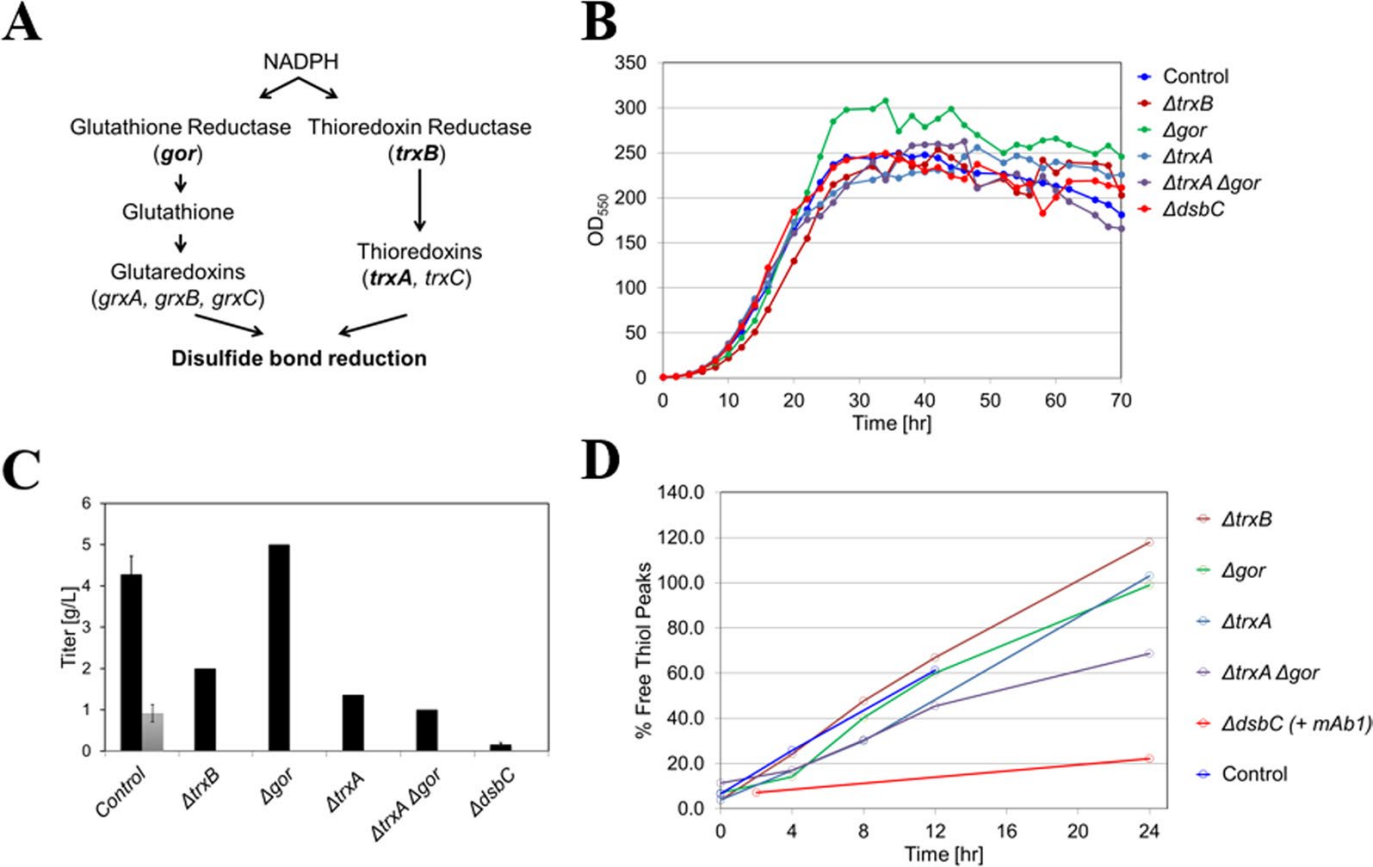
mAb1 fermentations using mutants lacking enzymes in thiol redox pathways. **a** Thiol-redox pathways in *E. coli* cytoplasm (selected mutants shown in bold). **b** Growth, **c** Titer (product titer in black bars and DsbC titer (control case) in grey bar) **d** Free thiol content during homogenate hold. The Δ*trxA*, Δ*trxB*, Δ*gor*, and Δt*rxA* Δ*gor* mutants contain pmAb1 plasmid coding for mAb1 and also overexpressing DsbA and DsbC. The ΔdsbC mutant contains pRM1 plasmid (pmAb1 without the *dsbC* gene). For determining free thiol formation in homogenate derived from ΔdsbC mutant fermentation, mAb1 was spiked at 4 mg/mL final concentration. OD_550_ results shown for control and Δ*dsbC* cases are average of n = 3 runs. Titer results shown for control and Δ*dsbC* cases are average of n = 3 runs and 1 s.d. For Δ*trxB,* Δ*trxA,* Δ*gor,* and Δ*trxA* Δ*gor* cases, OD_550_ and titer results shown are for n = 1 run. RP-HPLC assay results are for n = 1 sample

One of the major enzymatic reducing pathways of *E. coli* is the thioredoxin system (Fig. 6a). It has been shown that IgGs can be a substrate for the thioredoxin system [10] and recombinant antibodies can undergo thioredoxin-mediated reduction during production in CHO cells [11–13]. Therefore, mAb1 homogenate was incubated in the presence or absence of ebselen, a known inhibitor of *E. coli* thioredoxin reductase [14]. Ebselen completely prevented free thiol formation in mAb1 during the homogenate hold for up to 48 h at 25 °C (Fig. 5c), thus further confirming enzymatic basis of the observed free thiol formation.

### Genetic studies to identify enzyme(s) involved in free thiol formation in homogenate

In *E. coli*, the thioredoxin and glutaredoxin pathways are responsible for reduction of disulfide bonds in cytoplasmic proteins formed during their catalytic or regulatory cycles (Fig. 6a) [15, 16]. Several mutants were constructed by deleting one or more genes in the thioredoxin and glutaredoxin pathways in the mAb1 production host background (∆*trxB*, ∆*trxA,* ∆*gor,* ∆*trxA* ∆*gor*) to identify the enzyme(s) that are involved in reducing disulfide bonds in mAb1 during homogenate hold step.

Fermentations were performed using the thioredoxin and/or glutaredoxin pathway deficient mutants and homogenate derived from these fermentations were held for up to 24 h to understand whether the mutants prevented free thiol formation in mAb1. While growth was comparable among the different mutants, product titers of mutant strain fermentations (except for Δ*gor* mutant strain fermentation) were much lower than control strain fermentation (Fig. 6b, c). Levels of DsbC (0.91 g/L corresponds to approximately 39 mM) on a molar basis were similar to the levels of mAb1 (4.28 g/L corresponds to approximately 43 mM) produced in the control strain fermentation (Fig. 6c). When analyzed for free thiol content after homogenate hold, the single mutants (∆*trxB*, ∆*trxA*, and ∆*gor*) had similar rate of free thiol formation as the control strain during the homogenate hold step in spite of the lower product concentration for some cases (Fig. 6d). The ∆*trxA* ∆*gor* mutant had a modest decrease in the rate of free thiol formation likely due to the lower product concentration in the homogenate. These results indicate enzymes in thioredoxin or glutaredoxin pathways may not be directly responsible for increase in free thiol content in mAb1 during homogenate hold.

**Fig. 7:**
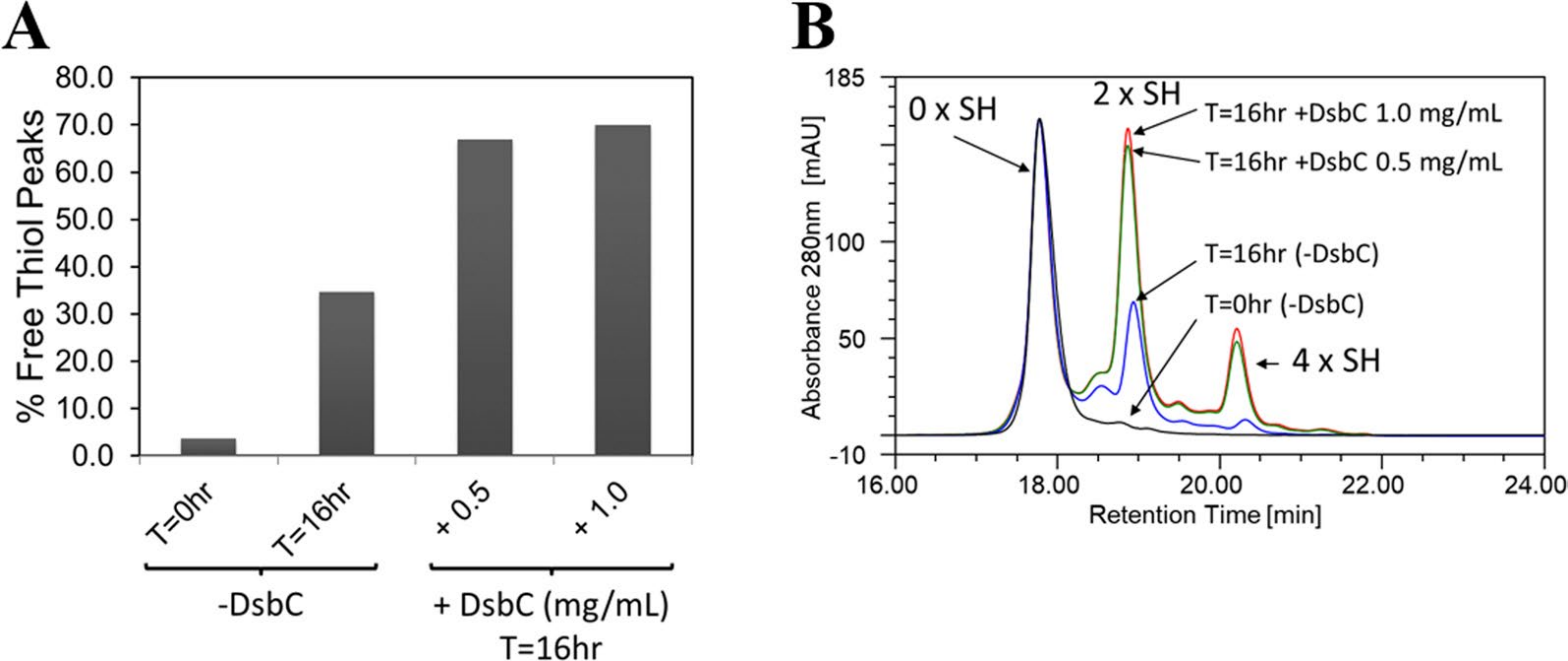
DsbC is responsible for disulfide bond reduction in mAb1 during blank host homogenate hold. A blank host fermentation was carried out using the same production host and process, but the plasmid did not contain genes for expression of mAb1 light and heavy chains and *dsbC*. **a** Dose dependent increase in free thiol in presence of DsbC. Note that “-DsbC” indicates that no DsbC was spiked. A blank host homogenate contains DsbC due to chromosomal expression, **b** RP-HPLC profiles of mAb1 MSS pools derived from homogenate held for 16 h in presence of DsbC. Results shown are for n = 1 sample

Disulfide bond isomerase C (DsbC) is overexpressed during the fermentation process to promote correct folding and assembly of mAb1 [17]. A mutant strain deleted in *dsbC* was evaluated to understand whether this enzyme is involved in reduction of disulfide bonds in mAb1 during homogenate hold. The ∆*dsbC* mutant fermentation performed poorly (like the thioredoxin pathway deleted mutants) and resulted in very low product titers, as expected, since this enzyme is required for correct folding and assembly of mAb1. To circumvent the low product titers, mAb1 was spiked into the ∆*dsbC* mutant homogenate at a final concentration of 4 mg/mL (typical end of fermentation titers in whole broth from a control fermentation) and the rate of free thiol formation was found to be significantly reduced (Fig. 6d). In addition, spiking recombinant DsbC (0.5–1.0 mg/mL) and mAb1 into the homogenate derived from the blank host fermentation (blank host fermentation was carried out using the same production host and process, but the plasmid did not contain genes for expression of mAb1 light and heavy chains, DsbA, and DsbC) a resulted in an evident increase in total free thiol content of mAb1, indicating DsbC is involved in free thiol formation in mAb1 during the homogenate hold step (Fig. 7a). Note that the blank host fermentation had chromosomal expression of DsbC which likely caused an increase in free thiols in mAb1 during the homogenate hold step (Fig. 7a). Similar to DS materials and homogenate hold-increased free thiol content, DsbC-induced free thiol content was predominantly due to the increased amount of mAb1 species with 2 unpaired cysteine residues and to lesser extent species with 4 unpaired cysteine residues (Fig. 7b).

### Impact of Fc glycosylation on the susceptibility to free thiol formation in homogenate

The remarkable site selective enzymatic reduction of the mAb1 C_H_2 domain disulfide bond prompted further experiments to understand factors governing it. mAb1 is naturally aglycosylated and lacks Fc N-glycans in the C_H_2 domain, presence of which is a conserved feature of IgG antibodies. We hypothesized that the lack of glycans may be a cause of the C_H_2 domain susceptibility to free thiol formation. Therefore, we examined whether antibody with the Fc glycans would be resistant to free thiol formation and removal of glycans can confer susceptibility to free thiol formation during the homogenate hold.

**Fig. 8:**
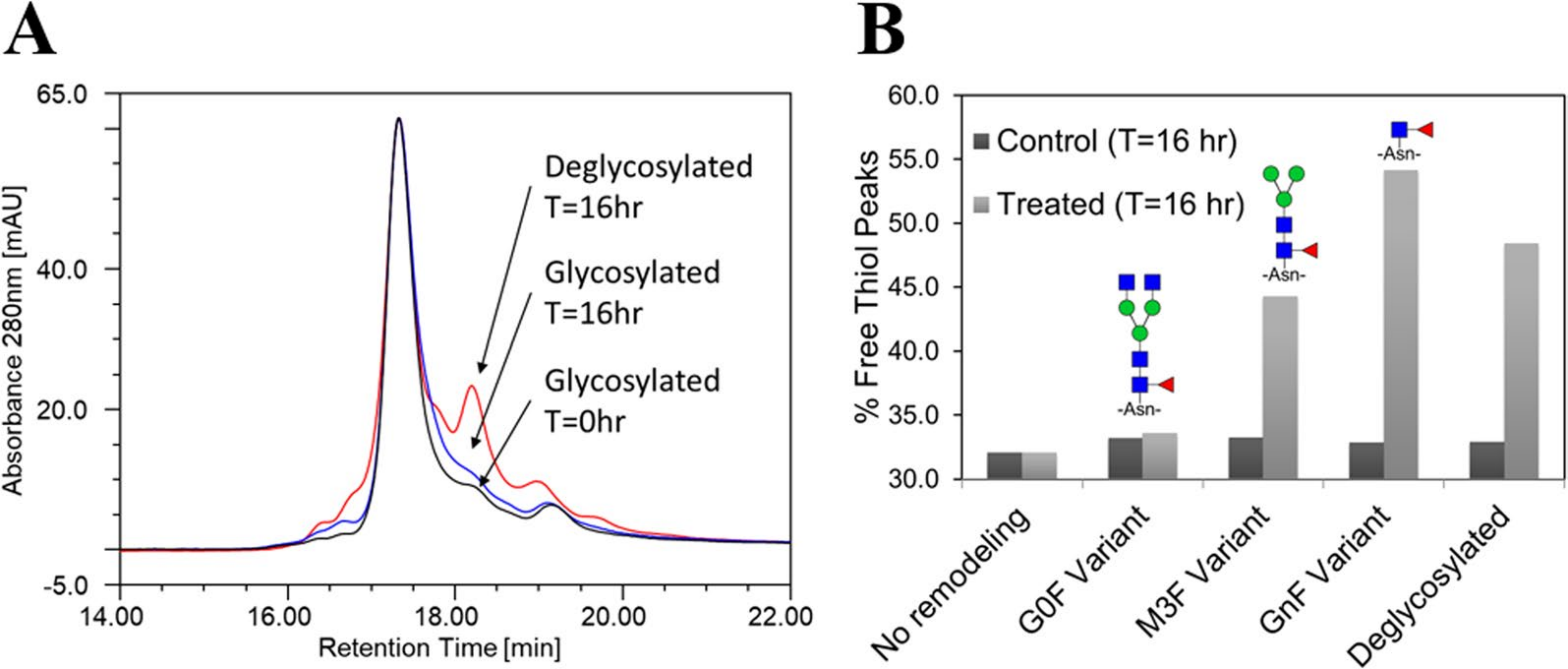
Effect of Fc glycosylation status on free thiol formation. **a** RP-HPLC profiles of glycosylated and de-glycosylated mAb2 MSS pools derived from homogenate held for 16 h **b** Homogenate hold-induced free thiol levels in fully deglycosylated mAb2 or mAb2 with enzymatically trimmed glycans. Results shown are for n = 1 sample

Not having the glycosylated version of mAb1, we used mAb2 (produced in CHO cells), a bivalent IgG1 with typical Fc glycosylation and the framework largely similar to mAb1. The C_H_2 domain amino acid sequences are identical between mAb1 and mAb2. Glycosylated or PNGase F-deglycosylated mAb2 was spiked into the homogenate derived from the blank host *E. coli* fermentation and total free thiol content was determined upon incubation (16 h at 25 °C). There were no major qualitative changes in the pattern of free thiol peaks and no increase of free thiols for the glycosylated mAb2. On the other hand, profile of the deglycosylated mAb2 was indicative of the increased free thiol content (Fig. 8a), and free thiols increased by ~ 16% compared t = 0 control. Next, Fc glycosylation of mAb2 was remodeled in vitro to generate variants with the progressively truncated glycans (predominantly G0F, M3F, and GnF glycoforms) in order to determine whether the Fc glycan size impacts susceptibility to free thiol formation. Variants and controls (mAb2 without glycan remodeling and deglycosylated mAb2) were spiked into the homogenate derived from the blank host fermentation and extent of free thiol formation was determined upon 16 h 25 °C hold. mAb2 without glycan remodeling and G0F variant (decrease in glycan size due to removal of only galactose residues) did not show appreciable free thiol formation. On the other hand, M3F and GnF variants (substantial and near complete glycan size reduction, respectively) showed increasing free thiol formation with the decreasing size of the Fc glycans (Fig. 8b), indicating that truncation of mAb2 Fc glycans resulted in the increased susceptibility to the free thiol formation in homogenate.

## Discussion

We identified the presence of free thiols and total free thiol content variability in a recombinant monovalent one-armed IgG1 antibody (mAb1) expressed in *E. coli*. Observed free thiol content variability of mAb1 DS materials was due to the variable levels of species with predominantly two unpaired intra-chain cysteine residues in the C_H_2 domain. This distribution is different compared to distribution of free thiols among IgG domains of several CHO-expressed recombinant IgGs, where the C_H_3 domain had the highest level of free thiols followed by C_H_1, C_H_2 and the variable domain in the heavy chain [18]. It is also distinct from reported more unique cases of free thiols corresponding to intra-chain cysteine residues in the heavy chain variable domain [19, 20]. The intra-chain location of free thiols in the C_H_2 domain (where disulfide bond would normally be buried between two anti-parallel β-sheets and would not be solvent exposed) suggested decreased reactivity [18]. This is consistent with our observations that Fc free thiols were not accessible to thiol-reactive Ellman’s reagent under non-denaturing conditions (levels were below limit of quantitation, ~ 0.1 mol free thiols/mole protein, of the assay), were not susceptible to in vitro re-oxidation, and remained stable during long-term storage of mAb1 drug product.

Experiments to identify the process step(s) responsible for free thiol formation determined that production culture and downstream purification did not contribute to variability of the free thiol levels and that one or more of the harvest steps was a culprit. We demonstrated that the longer hold time and increased temperature of *E. coli* homogenate can lead to the increase in total free thiol content of mAb1. The location of these induced free thiols predominantly in the C_H_2 domain, matched that of DS materials, thus corroborating that free thiol variability of DS materials was likely introduced when the product was in the homogenate. Additional information of high practical importance was obtained when we determined that flocculation, which removes insoluble cell debris and host cell proteins, completely blocked free thiol formation. This indicated that more stringent process control of the homogenate (hold time and temperature) prior to flocculant addition should be able to minimize mAb1 free thiol content, as well as decrease its variability. Indeed, subsequently designed and implemented process enhancements controlling those parameters resulted in low free thiol content of DS materials and ensured improved control over the consistency of free thiol levels in mAb1 produced at manufacturing-scale.

Mechanistically, the ability of the homogenate to induce Fc free thiols suggested that free thiols in DS materials were not the result of incomplete disulfide bond formation during assembly of mAb1, but rather that they were caused by the subsequent reduction of disulfide bonds. A dialysis experiment indicated that the mAb1 disulfide bond reduction in homogenate was enzyme mediated, and thus genetic studies were designed to identify enzyme(s) that reduce mAb1 disulfide bonds in the homogenate. Thioredoxin and glutaredoxin pathways are two well-known pathways responsible for the reduction of disulfide bonds in the cytoplasm of wild-type *E. coli* [21]. Inhibition of free thiol formation by the ebselen, a known competitive inhibitor of thioredoxin reductase (*trxB* gene product) [14], initially suggested involvement of thioredoxin pathway. However, single mutants of thioredoxin pathway (∆*trxB*, ∆*trxA*) did not show a decrease in the rate of free thiol formation, indicating thioredoxins may not be involved. Similarly, the glutaredoxin pathway mutant, ∆gor, also did not result in a decrease in the rate of free thiol formation. Modest effect of the double mutant (∆*trxA* ∆*gor*) was likely due to the low mAb1 concentration in homogenate, resulting in the lower rate of the enzymatic reaction. This apparent lack of impact from both reducing pathways seemed surprising; however, it appeared to be consistent with the observed selectivity of homogenate reduction for the intra-chain disulfide bonds rather than inter-chain bonds (hinge region and heavy chain-light chain disulfides). *E. coli* and mammalian thioredoxins (structurally similar to bacterial thioredoxins), can reduce more easily accessible inter-chain disulfide bonds in IgGs and not intra-chain disulfides [10, 11]. Similarly, glutaredoxins, the glutaredoxin pathway’s terminal enzymes also belonging to thioredoxin fold class, have been implicated in reduction of inter-chain disulfide bonds of IgGs expressed in CHO cells [13].

The apparent lack of involvement of thioredoxin and glutaredoxin pathways shifted our focus to the disulfide bond isomerase C (DsbC), one of the two periplasmic enzymes over-expressed to promote correct folding and disulfide bond formation in mAb1. DsbC is soluble homodimeric protein with two C-terminal catalytic domains containing the thioredoxin fold [22]. DsbC can function as disulfide bond isomerase responsible for rearranging incorrectly formed (non-native) disulfide bonds [4, 23–25] introduced by DsbA, which catalyzes rapid formation of disulfide bonds in newly synthesized proteins [3, 26]. Over-expression of DsbC is required for proper folding of proteins with multiple non-consecutive disulfide bonds [27] and it can improve yields of recombinant proteins containing multiple disulfide bonds [28]. Interestingly, rate of free thiol formation was significantly reduced in the homogenate derived from the ∆*dsbC* mutant fermentation. We were also able to demonstrate that recombinant DsbC spiked into homogenate derived from the blank host fermentation caused an increase in mAb1 free thiols compared to control (where some level of free thiol induction was observed, possibly due to expression of chromosomal *dsbC*). In addition, HPLC pattern of DsbC-induced free thiol species was consistent with that of DS materials. Altogether this was intriguing, since it indicated that DsbC is involved in homogenate’s ability to reduce disulfide bonds in mAb1. These results also imply that Ebselen could be directly or indirectly inhibiting DsbC and thus preventing free thiol formation in the homogenate as shown in Fig. 5c.

In *E. coli* cells, compartmentalization ensures separation of the reducing environment of cytoplasm from the oxidative environment of periplasm required for the proper formation of disulfide bonds [29]. It was clear that in intact cells, DsbC effectively engages mAb1 as substrate in the oxidative protein folding process occurring in the periplasmic space, since without over-expression of DsbC, very low titers of mAb1 were obtained. Lack of increase in free thiols during the whole cell broth hold indicated that already folded mAb1 (residing in the periplasm) is not prone to reduction, suggesting an inability to effectively engage the reducing enzyme activity observed in the homogenate. On the other hand, homogenization destroys cell compartmentalization, which results in non-native redox environment maintaining released components of both cytoplasm and periplasm under reducing conditions. Under such conditions, DsbC might be able to re-engage with mAb1 leading to the selective Fc disulfide bond reduction. Besides acting as disulfide bond isomerase, DsbC can also function as a disulfide reductase, where the reaction results in the reduction of the substrate and the oxidation of DsbC [29] instead of disulfide bond reshuffling. Increasing evidence appears to suggest that this mode of action, resulting in reduced substrate which can be then reoxidized by oxidants like DsbA, might be more relevant than true isomerase activity of DsbC [29]. Thus, reductase activity of DsbC could be causing disulfide bond reduction of mAb1 and relatively more reducing potential of homogenate (compared to the periplasm) might be preventing formation of those disulfide bonds. If DsbC is reducing the mAb1 C_H_2 disulfide bonds, what enzyme is responsible for reducing and thus recycling the oxidized DsbC? DsbD, a membrane protein, is the physiological reductant of DsbC inside cells [23]. However, DsbD is unlikely to be active in homogenate due to lack of membranes. Genetic studies with thiol redox pathway mutants indicate that the thioredoxin and glutaredoxin pathways may not be involved in mAb1 disulfide bond reduction in the homogenate. We speculate that DsbC reduction is not needed for free thiol formation in homogenate since the levels of DsbC on a molar basis are similar to the level of mAb1 in homogenate (Fig. 6c, control case). Such high levels of DsbC are sufficient for disulfide bond reduction in mAb1 in the homogenate without the need for its recycling.

Homogenate-induced reduction is remarkably selective towards the disulfide bond buried within C_H_2 domain and this poses a question how DsbC can re-engage with folded mAb1 and achieve such site-specific reduction. The answer could be a chaperone activity that DsbC also exhibits, and which is thought to be important for its function as an isomerase [30], since incorrectly formed disulfide bonds often might be buried within misfolded protein. DsbC can interact with folded proteins like for example AraF, where DsbC was shown to reduce poorly accessible single cysteine residue buried in the cleft of the protein [31]. In IgGs, the C_H_2 domains are the only unpaired domains. However, they contain conserved *N*-glycosylation site (Asn-297), occupied by glycans which extend into the Fc cavity and can make contacts with the protein surface [32, 33], thus making it potentially less accessible. On the other hand, mAb1 lacks Fc glycans and its Fc cavity surface (with exposed hydrophobic patches) may be more accessible to DsbC (or other chaperones), thus potentially allowing it to re-engage as chaperone and reductase. This would be consistent with our observation that presence of Fc glycans in mAb2 prevented, while their removal or trimming conferred susceptibility to free thiol formation during homogenate hold.

There might be additional structural basis for the selectivity towards the C_H_2 domain. In *E. coli* periplasm, DsbC is maintained exclusively in catalytically active reduced state by inner membrane protein DsbD [34–38]. Interestingly, DsbD belongs to a small subset of *E. coli* proteins containing immunoglobulin-like domains [39]. DsbD-α, a periplasmic N-terminal domain of DsbD with thiol oxidoreductase activity, forms a complex with DsbC and reduces it. This DsbD-α domain has classical c-type Ig fold structure [40, 41], therefore, maintenance of the normal function of DsbC in periplasm relies on the recognition and interaction with the Ig fold-based protein domain. Since, IgG C_H_2 domain shares structural similarity with DsbD-α due to presence of c-type Ig fold (Additional file 1: Fig. S1), we hypothesize that in homogenate, re-engagement of DsbC with the C_H_2 domain of mAb1 could be facilitated by recognition of the Ig fold structure and chaperone-substrate like interactions due to lack of Fc glycans. This could explain selective disulfide bond reduction predominantly in the C_H_2 domain of mAb1 and absence of significant free thiol formation in the Fab. Further work is needed to examine this hypothesis and to show direct interaction of DsbC with IgG1 Fc.

## Conclusions

*Escherichia coli* offers several advantages as a production host for manufacturing of recombinant proteins including antibody fragments. Controlling critical product quality attributes within a desired range is essential for antibody manufacturing processes especially for those antibodies used for therapeutic applications. Here we describe significant variability in the degree of disulfide bond reduction and consequently the total free thiol content in a one-armed antibody produced in *E. coli*. After further investigation, we conclude that the antibody disulfide bond reduction is catalyzed by the enzyme DsbC and that this reduction occurs in the homogenate. Our results also indicate that this disulfide bond reduction primarily occurs in the Fc C_H_2 region and lack of glycans in *E. coli* produced antibodies may result in this region being more accessible for redox enzymes like DsbC. In addition, we discuss some solutions such as minimizing homogenate hold times, reducing homogenate temperature during downstream processing, using enzyme inhibitors, adding flocculants prior to homogenization, or modulating redox environment in the homogenate to minimize disulfide bond reduction and better control antibody product quality attributes such as total free thiol content.

## Methods

### Bacterial strains, plasmids, and oligonucleotides

All strains evaluated in this study are derivatives of W3110 [42]. All strains and plasmids used in this study are listed in Table 1. Antibiotic selection was maintained for all markers at the following concentrations: Carbenicillin (plasmid or chromosomal), 50 μg/mL; Kanamycin (chromosomal), 30 μg/mL; Tetracycline (plasmid or chromosomal), 20 μg/mL.

**Table 1:** Bacterial strains and plasmids used in this study

Strain	Genotype or description	Source
W3110	F^−^ λ^−^ IN(*rrnD-rrnE*) *rph-1 ∆fnr*	Laboratory collection
68A8	Δ*fhuA* Δ*phoA ilvG2096 (*IlvG^+^, Val^r^*)* Δ*manA* Δ*trxA14::kan*	Laboratory collection
JW0871-1	F^−^, Δ*(araD-araB)567*, Δ*lacZ4787*(::rrnB-3), *λ*^*−*^, Δ*trxB786::kan*, *rph-1*, Δ*(rhaD-rhaB)568*, *hsdR514*	Coli Genetic Stock Center (CGSC)
JW3467-1	F-, Δ*(araD-araB)567*, Δ*lacZ4787*(::rrnB-3), *λ*^*−*^, Δ*gor756::kan*, *rph-1*, Δ*(rhaD-rhaB)568*, *hsdR514*	CGSC
JW2861-1	F-, c*(araD-araB)567*, Δ*lacZ4787*(::rrnB-3), *λ*^*−*^, Δ*dsbC744::kan*, *rph-1*, Δ*(rhaD-rhaB)568*, *hsdR514*	CGSC
64B4	W3110 ∆*fhuA* ∆*phoA ilvG2096* (IlvG^+^; Val^r^) ∆*manA* ∆*prc spr43H1* ∆*degP lacI*^*q*^ ∆*ompT*	Laboratory collection
68B1	64B4 Δ*trxA14::kan*	This study
68A6	64B4 Δ*trxB786*::*kan*	This study
68A7	64B4 Δ*gor756*::*kan*	This study
68B9	64B4 Δ*gor756*	This study
68C1	64B4 Δ*trxA14::kan* Δ*gor756*	This study
68D3	64B4 Δ*dsbC744::kan*	This study
68D5	64B4 Δ*dsbC744*	This study
Plasmid		
pmAb1	pBR322 plasmid coding for mAb1 and also overexpressing DsbA and DsbC	Laboratory collection
pRM1	pmAb1 without the *dsbC* gene	This study

### Strain and plasmid construction

Standard techniques were used for PCR, transformation, electroporation, and P1 transduction. All chromosomal modifications were confirmed by PCR and DNA sequencing.

The Δ*trxA::kan*, Δ*trxB::kan*, Δ*gor::kan*, Δ*dsbC::kan* alleles were separately introduced into 64B4 by P1 transduction from the strains 68A8, JW0871-1, JW3467-1, and JW2861-1 respectively to obtain 68B1, 68A6, 68A7, and 68D3 respectively. The kanamycin resistance marker from the 68A7 and 68D3 strains were removed using the pCP20 plasmid [43] to obtain the strains 68B9 and 68D5 respectively. To construct the double mutant 68C1, the Δ*trxA::kan* allele was introduced into 68B9 by P1 transduction.

Expression cassettes coding for light and heavy chains of mAb1, *dsbA*, and *dsbC* were cloned into pBR322 as described [44] to obtain the plasmid pmAb1. The *dsbA* and *dsbC* genes in pmAb1 was first removed by digesting pmAb1 with AatII and PvuI. The *dsbA* gene was PCR amplified from pmAb1 using primers AatII-dsbA-F (5ʹ-TATGACGTCCATGCTGTGGTGTCATGGTCGG-3ʹ) and PvuI-dsbA-R (5ʹ-TATCGATCGGGTACCTTATTTTTTCTCGGAC-3ʹ) and ligated into AatII and PvuI digested pmAb1 to obtain the plasmid pRM1.

### Fermentation, harvest, and purification processes

Fermentations were performed as described [45]. A blank host fermentation was carried out using the same production host and process, but the plasmid did not contain genes for expression of mAb1 light and heavy chains, DsbA, and DsbC. Recombinant protein harvest and purification was performed as described previously [1] with minor modifications.

### Analytical measurements

For product titer measurements, fermentation broth samples were diluted 1:10 with extraction buffer (10 mM Tris, 5 mM EDTA, 5 mM iodoacetamide (IAM), 0.2 mg/mL lysozyme, pH 6.8), lysed by sonication and centrifuged at 17,000×*g* for 20 min at 4 °C. Product titer was determined from supernatants using reversed-phase high performance liquid chromatography (RP-HPLC). For DsbC measurements, fermentation broth samples were diluted tenfold in denaturing buffer (6M Guanidine-HCl/360 mM Tris-HCl, pH 8.6) and treated with DTT (100 mM), incubated at 60 °C for 20 min and centrifuged for 30 min at 17,000*g*. DsbC was determined from supernatants using RP-HPLC. Note that the sample derivatization and RP-HPLC methods used for product titer and DsbC measurements is different from the sample derivatization and RP-HPLC method used for free thiol analysis (described below). OD_550_ was measured using Genesys 20 visible spectrophotometer (Thermo Fisher Scientific) by measuring absorbance at 550 nm.

### Homogenate hold experiments

Scale of homogenate hold studies and protein purification was dependent on the purpose of the study or availability of the materials. Hold studies assessing effect of time, temperature, flocculant addition, ebselen (2-phenyl-1,2-benzisoselenazol-3(2H)-one; Sigma Millipore; final concentration 1 µM) were performed at 5 L scale. Homogenate hold studies with thiol redox pathway mutants were performed at 0.5 L scale. mAb1 was spiked into blank host-derived homogenate and the Δ*dsbC* mutant-derived homogenate to a final concentration of 4 mg/mL. *E. coli* whole cell broth was passed two times through a homogenizer and homogenate was held in closed containers in a temperature-controlled water bath. At the specified time points, homogenate was conditioned to a concentration of 0.8% PEI to promote flocculation and centrifuged to separate flocculated cell debris from supernatant. Supernatant was passed through a 0.2 µm filter and purified over MabSelect SuRe protein A resin (GE Healthcare) with an Akta Explorer FPLC (GE Healthcare).

Bench-scale homogenate hold model (1 mL homogenate in 2 mL screw cap tube) was used to assesses free thiol formation in glycosylation variants or to study the impact of recombinant DsbC, due to limited quantities of those materials. For DsbC spiking study, recombinant DsbC (produced in *E. coli* at Genentech) was added to a final concentration of 0.5 and 1.0 mg/mL. mAb2 glycosylation variants/remodeling treatment controls was spiked into blank host-derived homogenate to a final concentration of 1 mg/mL. Spiked homogenate was incubated for 16 h at 25 °C, conditioned with PEI at 30 °C for 30 min, and centrifuged (15 min, 14,000 rpm) to separate flocculated cell debris. mAb2 was affinity-purified from the supernatant using MabSelect SuRe protein A resin in a centrifugal spin format (200 μL resin; binding/wash buffer: 25 mM Tris-Cl, 25 mM NaCl, pH 7.7; elution buffer: 75 mM glycine phosphate, pH 3.3).

For the dialysis experiment, mAb1 (MSS pool) was placed inside a 5 kDa MWCO dialysis bag and the bag was placed in a container filled with homogenate derived from a blank host fermentation, and incubated up to 54 h at 25 °C. For control case, MSS pool was placed inside a closed container for up to 54 h at 25 °C. The MSS pool was then analyzed for free thiol levels.

### mAb2 glycosylation variants

Deglycosylated mAb2 was prepared using PNGase F digestion (N-Glycanase, ProZyme; overnight digestion in 10 mM ammonium formate pH 8.6, 37 °C). Glyco remodeled mAb2 variants were prepared by enzymatic digestion: with β1,4-galactosidase (New England Biolabs; G0F as predominant resulting glycoform); with β1,4-galactosidase followed by β-*N*-acetyl-hexosaminidase (Prozyme; M3F as predominant resulting glycoform); and with Remove-iT Endo-S endoglycosidase (New England Biolabs; GnF as predominant resulting glycoform). Respective treatment controls (no enzyme addition) were prepared in parallel. Deglycosylation and extent of enzymatic glycan remodeling were confirmed by intact and reduced mass LC–MS analysis (HPLC-Chip/TOF, Agilent Technologies).

### Free thiol analysis by RP-HPLC

Total free thiol content (accessible and buried free thiols) was determined by RP-HPLC with free thiol derivatization and UV detection as described [9]. Briefly, mAb MSS sample (harvest and purification process used as described before [1]) was diluted to 2 mg/mL with 20 mM sodium acetate buffer, pH 6.0. Diluted sample was mixed 1:1 v/v with denaturing/labeling reagent (8M guanidine hydrochloride/350 mM sodium acetate/202 µg/mL *N*-tert-Butylmaleimide) and incubated at 37 °C for 30 min. Sample was cooled down and analyzed by RP-HPLC using BEH300 C4 column (2.1 × 250 mm, 3.5 µm, 300 Ǻ; Waters Corporation). Chromatographic separation was performed at 70 °C (mAb1) or 80 °C (mAb2) using HPLC system (Agilent Technologies). The mobile phase A was 0.1% TFA in water and mobile phase B was 0.1% TFA in ACN (v/v). Initial conditions were set at 31% mobile phase B and kept for 1 min after sample injection. Concentration of mobile phase B was increased to 40% over next 29 min and then to 100% over 0.25 min and held for 2 min. Over next 0.25 min, concentration of mobile phase B was decreased back to 31% and for 12.5 min to re-equilibrate column. The flow rate for separation was 0.333 mL/min, while for wash step and first 7.5 min of the equilibration step flow rate was 0.5 mL/min. Protein detection was accomplished using UV detection at 280 nm. Free thiol content of the samples was expressed as % Free Thiol Peaks, where relative percent peak areas for peaks of interest (Main and Free Thiol peaks) were calculated using a weighted method for the free thiol portion (due to the weighted method for reporting free thiol levels, the total relative percent peak area for all peaks of-interest can be greater than 100%).

### Site-specific free thiol analysis by LC–MS peptide mapping

Site-specific free thiol quantitation was performed using differential *N*-ethylamaleimide (NEM) isotope tagging method developed at Genentech. Briefly, to tag accessible and buried free thiols in mAb with d0-NEM, 100 µL mAb (3 mg/mL) was mixed with 400 µL denaturing buffer (7.5 M GdnHCl, pH 5) containing 6.25 mM d0-NEM, and incubated at 37 °C for 2 h. Unreacted d0-NEM was inactivated by addition of 20 µL Cysteine (175 mM) followed by incubation at 37 °C for 15 min. Intact disulfides were reduced by addition of 10 µL TCEP (0.5 M) followed by incubation at 37 °C for 30 min. Free thiols corresponding to TCEP reduced disulfides were tagged by adding 70 µL d5-NEM (171 mM) and incubation at 37 °C for 2 h. Excess of reagents was removed from NEM tagged sample using buffer exchange on NAP-5 column (GE Healthcare), equilibrated and eluted (0.6 mL) with MOPS buffer (20 mM MOPS, 0.5 mM TCEP, pH 7). Eluted sample was digested with trypsin at 1:42 (w/w) trypsin:antibody ratio, at 37 °C for 2 h, and subsequently quenched with 10% TFA. Resulting peptides were separated on Jupiter C18 column (250 × 2 mm, 5 µm, 300 Å; Phenomenex) using an Agilent 1200 HPLC system (Agilent Technologies). 95 µL of the sample was injected on the column and chromatographic separation was performed at 55 °C. The mobile phase A was 0.1% TFA in water and mobile phase B was 0.08% TFA in 90% ACN (v/v). Initial conditions were set at 100% mobile phase A and kept for the first 3 min after sample injection. Mobile phase B was increased to 10% over the next 20 min and then further increased to 40% until 160 min, and 100% until 162 min. Mobile phase B was held at 100% until 170 min. The column was the re-equilibrated at 100% mobile phase A until 195 min. The flow rate was kept at 0.28 mL/min. The effluent from the HPLC was directly connected to the electrospray ionization source of LTQ Orbitrap mass spectrometer operating in a positive ion mode. The spray voltage was 4.5 kV, and the capillary temperature was 300 °C. The mass spectrometer was operated in the data dependent fashion to switch automatically between MS and MS/MS modes. Survey full scan MS spectra were acquired from m/z 300 to m/z 2000 in the FT-Orbitrap with a resolution set for R = 60,000 at m/z 400. The five most intense ions were fragmented in the linear ion trap using collision-induced dissociation (CID) at normalized collisional energy of 35% with an activation time of 30 ms and isolation width of 2.5 m/z units. The dynamic exclusion (DE) function was enabled to reduce data redundancy and allow low-intensity ions to be selected for data dependent MS/MS scans. The dynamic exclusion parameters were as follows: a repeat duration of 30 s, an exclusion list size of 500, an exclusion duration of 90 s, a low exclusion mass width 0.76, a high exclusion mass width of 1.56, and a repeat count of 2. The data analysis was performed using Xcalibur software (Thermo Fisher Scientific).

## Supplementary Information

**Additional file 1: Fig. S1.** Structural similarity of N-terminal domain (Ig fold) of thiol oxidoreductase DsbD (DsbD-α; PDB 1JPE; red) and C_H_2 domain (green) of the knob-into-hole Fc (PDB 4NQS; C_H_3 domain in grey). Only ½ of the Fc shown.

## Data Availability

All data generated or analyzed during this study are included in this article and its Additional file 1.

## Abbreviations

AEX: Anion exchange chromatography
CEX: Cation exchange chromatography
CHO: Chinese Hamster Ovary
CUFDF: Conditioned ultrafiltration and diafiltration
DS: Drug substance
DsbC: Disulfide bond isomerase C
DsbD: Disulfide bond isomerase D
*E.** coli*: *Escherichia coli*
FL: Flocculant
HH: Homogenate hold
kan: Kanamycin
IAM: Iodoacetamide
LC–MS: Liquid chromatography mass spectrometry
mAU: Milli absorbance units
MSS: MabSelect SuRe
MWCO: Molecular weight cut off
RP-HPLC: Reversed-phase high performance liquid chromatography
SH: Free thiol
UV: Ultraviolet
