# Supplementary figures and images for: Enzymatic basis of the Fc-selective intra-chain disulfide reduction and free thiol content variability in an antibody produced in Escherichia coli
Source: Microb Cell Fact. 2022 Aug 19;21:167. doi: 10.1186/s12934-022-01892-4 (PMC9392285; doi:10.1186/s12934-022-01892-4)

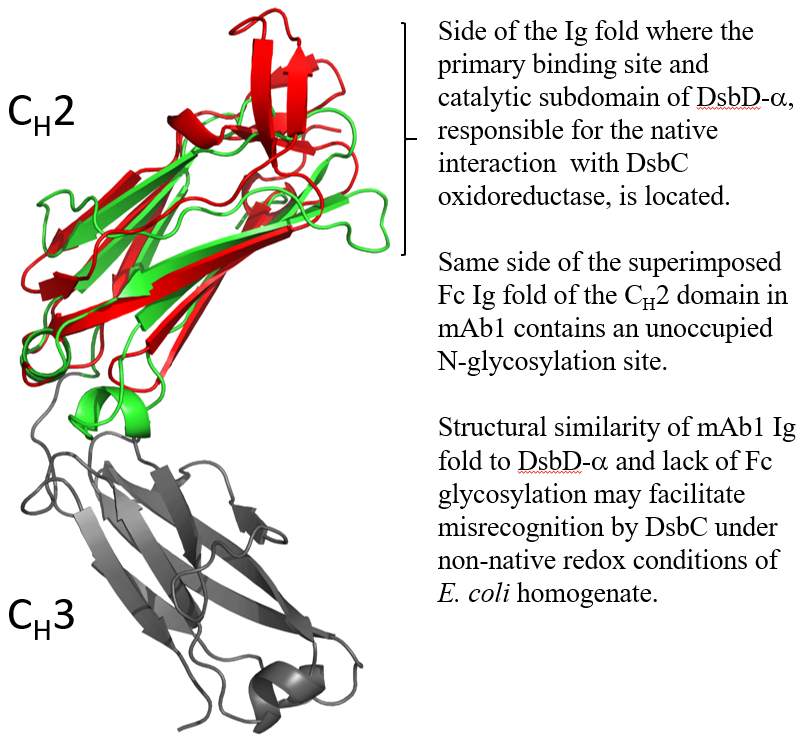

Supplement: Supplementary file 1 — Additional file 1: Fig. S1. Structural similarity of N-terminal domain (Ig fold) of thiol oxidoreductase DsbD (DsbD-α; PDB 1JPE; red) and CH2 domain (green) of the knob-into-hole Fc (PDB 4NQS; CH3 domain in grey). Only ½ of the Fc shown. [file 12934_2022_1892_MOESM1_ESM.tif]
